# Supplementary material for: The gap between knowledge and action in Zimbabwe: The limits of individual awareness in the face of structural violence in cholera endemicity
Source: PLoS One. 2026 Apr 21;21(4):e0347844. doi: 10.1371/journal.pone.0347844 (PMC13098957; doi:10.1371/journal.pone.0347844)
Supplement: S1 Table — (DOCX) [file pone.0347844.s001.docx]

QUESTIONARRIE

Dear Participant,

Thank you for taking the time to participate in this survey. Your input is invaluable to our research study titled " The Gap Between Knowledge and Action in Zimbabwe: The Limits of Individual Awareness in the Face of Structural Violence in Cholera Endemicity." The survey consists of several sections, including questions about your demographic information, and KAP. All questions are designed to be straightforward, and there are no right or wrong answers. Your responses will be kept completely confidential and will be used solely for academic research purposes. The survey should take approximately 10-15 minutes to complete.

Thank you again for your participation.

**Section A:** Demographic Characteristics

**Age: _____**

**Gender:** Male Female

**Level of education: ________**

**Occupation: _____________**

**Who are you living with? ______________**

**What is your place of residence? ______________**

**Section B:** KAP scale

| **KNOWLEDGE ON CHOLERA and WASH** | **YES** | **NO** |
| --- | --- | --- |
| Do you know watery diarrhea is a symptom of Cholera? |  |  |
| Do you know abdominal pain is a symptom of Cholera? |  |  |
| Do you know fever is a symptom of Cholera? |  |  |
| Do you know vomiting is a symptom of Cholera? |  |  |
| Do you know lethargy is a symptom of Cholera? |  |  |
| Do you know drinking contaminated water cause Cholera? |  |  |
| Do you believe contact with stool cause cholera? |  |  |
| Do you know eating contaminated food cause Cholera? |  |  |
| Do you know eating undercooked food cause Cholera? |  |  |
| Do you know that handling food with unclean hands can contribute to cholera transmission? |  |  |
| Do you know that severe dehydration is a major complication of cholera? |  |  |
| Do you know that if left untreated, cholera complications can lead to death? |  |  |
| Do you know washing hands frequently can prevent Cholera? |  |  |
| Do you know using sanitary latrines can prevent Cholera? |  |  |
| Do you know drinking treated water can prevent Cholera? |  |  |

| **ATTITUDE TOWARDS CHOLERA PREVENTION AND WASH** | **STRONGLY AGREE** | **AGREE** | **NEUTRAL** | **DISAGREE** | **STRONGLY DISAGREE** |
| --- | --- | --- | --- | --- | --- |
| Cholera is more severe than other diarrheal diseases. |  |  |  |  |  |
| I am concerned about the impact of cholera in my community. |  |  |  |  |  |
| I believe cholera is a curable disease if treated early. |  |  |  |  |  |
| I feel responsible for being involved in efforts to prevent and control cholera**.** |  |  |  |  |  |
| If I suspect cholera, I would take immediate action to prevent its spread. |  |  |  |  |  |
| If I or a family member had cholera symptoms, I would seek healthcare at a Cholera Treatment Center (CTC). |  |  |  |  |  |
| I would notify the nearest health center if I knew of a cholera case in my area. |  |  |  |  |  |
| If I or someone I know had cholera, I would seek treatment at a health center. |  |  |  |  |  |
| I believe traditional practitioners can effectively treat cholera |  |  |  |  |  |
| I would consider managing a cholera case at home instead of seeking medical help. |  |  |  |  |  |
| I believe prayer alone can cure cholera without medical treatment. |  |  |  |  |  |
| If someone had cholera, I would give Oral Rehydration Solution (ORS) at home before seeking medical help. |  |  |  |  |  |
| Do you always wash your hands with soap after using the toilet? | YES | NO | - | - | - |

| **PRACTICES RELATED TO WASH AND CHOLERA PREVENTION** | **YES** | **NO** |
| --- | --- | --- |
| Do you have a dedicated hand-washing facility at home? |  |  |
| Do all members of your household practice handwashing before eating or preparing food? |  |  |
| Do you always wash your hands with soap after using the toilet? |  |  |
| What is your main source of drinking water?  Select all that applies |  |  |
| Tap water |  |  |
| Lake |  |  |
| River |  |  |
| Improved dug well |  |  |
| Drilled borehole |  |  |
| Unimproved dug well |  |  |
| Do you treat your drinking water before consumption? |  |  |
| How do you treat your drinking water at home? Select all that applies |  |  |
| Chlorine/purifiers |  |  |
| Boiling |  |  |
| Filtation |  |  |
| No treatment |  |  |
| How do you store drinking water at home? |  |  |
| Covered container |  |  |
| Uncovered Container |  |  |
| What type of latrine do you use at home? |  |  |
| Pour-flush latrine |  |  |
| Latrine connected to septic tank |  |  |
| Uncovered simple pit latrine |  |  |
| Covered simple pit latrine |  |  |
| Latrine dumped in an unknown location |  |  |
| How does your household dispose of human waste? |  |  |
| Latrine |  |  |
| Open defecation |  |  |
| Burial |  |  |
| Other |  |  |
