# Supplementary material for: The gap between knowledge and action in Zimbabwe: The limits of individual awareness in the face of structural violence in cholera endemicity
Source: PLoS One. 2026 Apr 21;21(4):e0347844. doi: 10.1371/journal.pone.0347844 (PMC13098957; doi:10.1371/journal.pone.0347844)
Supplement: Attachment — Submitted filename: Comment_TM.docx [file pone.0347844.s002.docx]

**Comments:**
This is a well‑written article that clearly describes the current cholera situation on the ground. The manuscript effectively highlights that the ongoing outbreak is largely driven by the lack of adequate WASH (Water, Sanitation, and Hygiene) facilities. The contextual explanations are strong and help the reader understand the underlying structural challenges contributing to transmission.

**Minor Edits:**

1. The correct scientific name is *Vibrio cholerae*, not *Vibrio cholera*. Please update this throughout the manuscript.
