# Supplementary material for: The gap between knowledge and action in Zimbabwe: The limits of individual awareness in the face of structural violence in cholera endemicity
Source: PLoS One. 2026 Apr 21;21(4):e0347844. doi: 10.1371/journal.pone.0347844 (PMC13098957; doi:10.1371/journal.pone.0347844)
Supplement: Attachment — Submitted filename: Response to Reviewers.docx [file pone.0347844.s003.docx]

**Response to Reviewers**

**Manuscript ID:** PONE-D-26-12022

**Title:** The Gap Between Knowledge and Action in Zimbabwe: The Limits of Individual Awareness in the Face of Structural Violence in Cholera Endemicity

**Authors:** Saime Ulucayli, Afet Arkut, Anele Lunga

Dear Dr. Parker and the Editorial Board of PLOS ONE,

We would like to thank the editorial team and the reviewers for their insightful comments and constructive feedback. We have addressed each point raised and believe the revisions have significantly strengthened the manuscript. All changes are highlighted using the **Track Changes** feature in the revised manuscript.

Below is our point-by-point response:

**Part 1: Academic Editor’s Requirements**

**1. Title and Header Correction:**

- **Comment:** Change your title from "To the Editorial Board, PLOS ONE..." to the actual title.
- **Response:** We have removed the editorial salutation from the header. The manuscript now begins directly with the formal title.

**(See Lines 1-3)**

**2. Data Availability Statement:**

- **Comment:** Address restrictions to data sharing.
- **Response:** In compliance with PLOS ONE’s policy, we have provided the minimal, fully anonymized data set as a CSV file (**S1_Data.csv**) under the Supporting Information category. This includes all raw data used for the statistical analyses.

**(See Data Availability Statement, Lines 394-396)**

**3. Ethics Statement Placement:**

- **Comment:** Ethics statement should only appear in the Methods section.
- **Response:** The ethics statement has been removed from the end of the manuscript and is now exclusively located within the Methods section under the "Ethical Approval" subsection.

**(See Lines 127-134)**

**Part 2: Response to Reviewer #1**

**1. Scientific Nomenclature:**

- **Comment:** Use the correct name *Vibrio cholerae*.
- **Response:** We have corrected "Vibrio cholera" to ***Vibrio cholerae*** (italicized) throughout the entire manuscript.

**(See Lines 33, 35, 41, 42 and 44)**

**Part 3: Response to Reviewer #2**

**1. Grammar and Opening Sentence:**

- **Comment:** The first sentence of the introduction needs attention.
- **Response:** We have rewritten the first sentence to correct the grammar and to better introduce the biological and structural context of the study.

**(See Lines 33-43)**

**2. 'Behaviour Change' Narrative and Human Rights:**

- **Comment:** Strengthen the argument that the ‘behaviour change’ narrative has little value in infrastructure-deprived environments.
- **Response:** We have revised the Introduction to emphasize that WASH infrastructure is a fundamental human right. We explicitly state that focusing on individual behavior without addressing structural deprivation can be counterproductive.

**(See Lines 33-43)**

**3. Study Population Rationale (Education and Internet):**

- **Comment:** Clarify why a sample with high education and internet access was examined.
- **Response:** We have added a clarification in the Methods section. We argue that this sample provides an "analytical advantage": it allowed us to examine whether individuals who possess both the education and the means to access health information are still constrained by systemic factors. By focusing on this group, we were able to isolate the impact of structural violence from individual awareness, demonstrating that even high health literacy cannot overcome the barriers of a decaying municipal infrastructure.

**(See Lines 106-116)**

**4. Oral Cholera Vaccines (OCV):**

- **Comment:** Address why vaccinations are not referred to.
- **Response:** We have integrated a discussion on OCV, citing **Hichilema and Ghebreyesus (2025)**. We clarify that while vaccines are vital reactive tools, our focus is on the more sustainable solution of long-term infrastructural investment.

**(See Lines 299-305)**

**5. Qualitative Literature (Oberg, 2019):**

- **Comment:** Refer to qualitative research like Oberg (2019).
- **Response:** We have incorporated **Oberg (2019)** into the Discussion to support our findings regarding the failure of individual-focused behavior change messaging in the absence of basic services.

**(See Lines 255-258)**

**6. Table 1 "Other" Category:**

- **Comment:** Clarify the "Other" gender category.
- **Response:** We have added a clarifying note below Table 1, specifying that this category includes participants who chose not to self-identify or preferred not to disclose their gender.

**(See Lines 183-185)**

We hope these revisions meet the requirements for publication in **PLOS ONE**.

Sincerely,

**Dr. Saime Ulucayli** (on behalf of all authors)

*Corresponding Author* Cyprus International University
